# Instituting a Green Zone for Elective Surgery During the Second Wave of COVID-19

**DOI:** 10.7759/cureus.19584

**Published:** 2021-11-15

**Authors:** Muhammad Rafaih Iqbal, Subiksha Subramonian, Kabir Matwala, Catherine Morrison, Stavros Karamanakos, Samer-ul Haque, Dennis Wayne Chicken, Bryony Lovett, Sarah-Jane Walton

**Affiliations:** 1 General Surgery, Mid and South Essex NHS Foundation Trust, Basildon, GBR

**Keywords:** morbidity, mortality, green zone, covid-19, elective surgery

## Abstract

Objective

Elective surgery came to a standstill during the first wave of COVID-19. The safe resumption of elective surgery with COVID-19 prevalent in the community remains a significant challenge. The aim of this study was to look into the outcomes of elective general surgery in a dedicated ‘Green Zone (GZ)’ during the second wave of COVID-19 in the United Kingdom.

Method

A ‘Green Zone’ pathway, meant to provide a COVID-free environment, was created. A retrospective review of prospectively collected data was done on consecutive patients who underwent an elective general surgical procedure at a single NHS trust over a six-month period (September 1, 2020, to February 28, 2021). The primary outcome was 30-day COVID-19 mortality. Secondary outcomes included 30-day non-COVID-19 mortality, readmissions, and complications.

Results

The study included 331 patients with a median age of 55 years (interquartile range, IQR, 41-67); 169 (51%) were females. The majority of the patients were American Society of Anaesthesiologists grade 2 (ASA 2; n=177, 53%) followed by ASA 3 (n=76, 23%). Forty-seven patients (14%) had been shielding earlier in the year. Most of the cases were day cases (n=224, 67%). There was no 30-day COVID-19 or non-COVID-19 mortality. One patient developed COVID-19 three weeks after the index operation. Thirty-day readmission and complication rate were 4% (n=14) and 6% (n=21). Most of the complications were Clavien-Dindo grade 2 (n=10, 3%) followed by an equal number of grades 1 and 3b (n=5, 1.5%).

Conclusion

This study has shown that a dedicated ‘Green Zone’ elective operating pathway is safe and feasible provided a balanced risk assessment approach is adopted.

## Introduction

COVID-19 pandemic changed the delivery of healthcare across the globe [1]. In the first wave of the pandemic, due to the increasing demand for emergency and Intensive Care Unit (ICU) services, elective activity came to a halt [2,3]. Approximately 28 million operations were cancelled globally during the peak 12 weeks of the first wave of the pandemic [4]. Data published reported a 51.2% rate of pulmonary complications in the perioperative period due to COIVID-19 and a mortality of 23.8% [5,6]. Non-urgent elective surgery was suspended for three months in the United Kingdom (UK) in April 2020 in order to accommodate patients requiring respiratory support [7].

In the UK, approximately 36,000 cancer surgeries were cancelled during the first wave. Clearing the backlog will require a minimum of 11 months with 20% extra activity at a cost of £2 billion [4]. Careful planning and strategy were required in order to restart the elective services [8]. Management strategies included COVID-free hospitals (cold sites) or a COVID-free area in acute settings (Green Zone, GZ) [9,10]. No one strategy suits all due to the regional variations in the COVID-19 infection rates and workload [11]. In addition, a phased return of the elective activity after the first wave was required. This was complicated by the reduced operating capacity as a result of enhanced infection control measures.

In response to this crisis, we created the concept of GZ at our hospital in order to restart the elective surgery. The GZ was a dedicated COVID-free area that could deliver surgical services in a safe way with no shared areas with acute services dealing with COVID-19 patients labelled as Red/Amber zones. This study aimed to assess the clinical outcomes of all the elective general surgical cases operated within the GZ during the second wave of COVID-19 in the UK.

## Materials and methods

Study design

This was a retrospective analysis of a prospectively maintained database of all the consecutive patients who underwent elective general surgery during the second peak of COVID-19 in the UK within a dedicated ‘GZ’ over a six-month period (September 1, 2020, to February 28, 2021).

Setting

Basildon University Hospital, in the East of England, serves a population of around 450,000 people living in and around South-West Essex. It has 25 inpatient wards and 637 inpatient beds with 12 operating theatres. It has a total of 126 surgical beds including a 16 bedded Day Surgery Unit (DSU).

After the cessation of the first wave of COVID-19, surgical services were reorganised in order to restart elective activity. The GZ was created as a COVID-19 protected area for elective surgery. DSU along with its three theatres and a nine-bedded adjoining ward, in addition to the 16 beds in DSU, was labelled as ‘GZ’. It was isolated from the acute services of the main hospital and had a separate entrance for patients and staff. The GZ had full access to the laboratory services including blood bank. Specific pathways were in place for the safe transfer of patients across to the radiology department if any imaging was required. Supplies from the main hospital were transferred to the GZ undertaking covid precautions.

Pathway

Each consultant reviewed and prioritised their waiting lists for patients requiring cancer surgery along with those requiring urgent non-cancer surgery according to the Royal Colleges [12] and National Institute for Health and Care Excellence (NICE) guidelines [13]. Patients were then telephoned and offered a date for surgery. Patients who agreed to surgery were asked to self-isolate for 14 days and report any COVID-19 symptoms in the 14 days prior to surgery. Preassessment was undertaken mostly by telephone. The need for a post-operative ICU bed for colorectal resections was evaluated on an individual case basis. If the probability was deemed high, alternative treatment options were considered. Pre-operative investigations such as phlebotomy and COVID-19 testing were undertaken at a non-acute site. All patients underwent COVID PCR swab testing 72 hours before surgery. A dedicated nurse specialist counter-checked patient notes at least 24 hours before the surgery. Patients undergoing a colorectal resection received pre-operative bowel preparation and oral antibiotics at home. All operating lists were reduced in order to maintain theatre protocol to mitigate the COVID-19 risk and also to avoid pressure on the limited beds' availability.

On the day of surgery, patients arrived in the DSU reception hall. Strict adherence to face masks and two metres social distancing was maintained. All patients and staff underwent a temperature check and were asked to fill in a questionnaire regarding their symptoms or contact with COVID-19 before entering the DSU. All the staff working in the GZ underwent lateral flow tests at least twice a week. Staff was asked to minimize contact with COVID-19 patients if working on the acute services. Both patient and staff questionnaires are available in the supplementary data appendix. No visitors were allowed within the GZ.

Standard national guidelines were followed in the operating theatre regarding personal protective equipment (PPE) [14,15]. All the patients were anaesthetised in the operating theatre with the anaesthetic team wearing full PPE. Once the patients were anaesthetised, the surgical and nursing team waited for 10 minutes in order to allow adequate air exchange to occur and to minimize the aerosol exposure. Upon completion of the surgery, a similar procedure was adopted at extubation with a wait of 10 minutes prior to transfer to the recovery. Recovery staff were appropriately trained in the management of arterial lines and central venous catheters if required. Enhanced recovery protocol was used in the post-operative management of all colorectal patients. When fully recovered all day cases were transferred to the DSU ward while the inpatients were transferred to the dedicated overnight stay ward.

All day cases were reviewed by the operating team before discharge. A consultant/senior registrar led ward round was conducted each day for the inpatients. Physiotherapy and stoma care nurses also undertook a ward round each morning. Each patient upon discharge was provided with a dedicated nurse specialist contact number in case required. All patients had a 30-day telephonic nurse-led follow up to evaluate their progress and to assess if they had any COVID-related symptoms.

**Figure 1:**
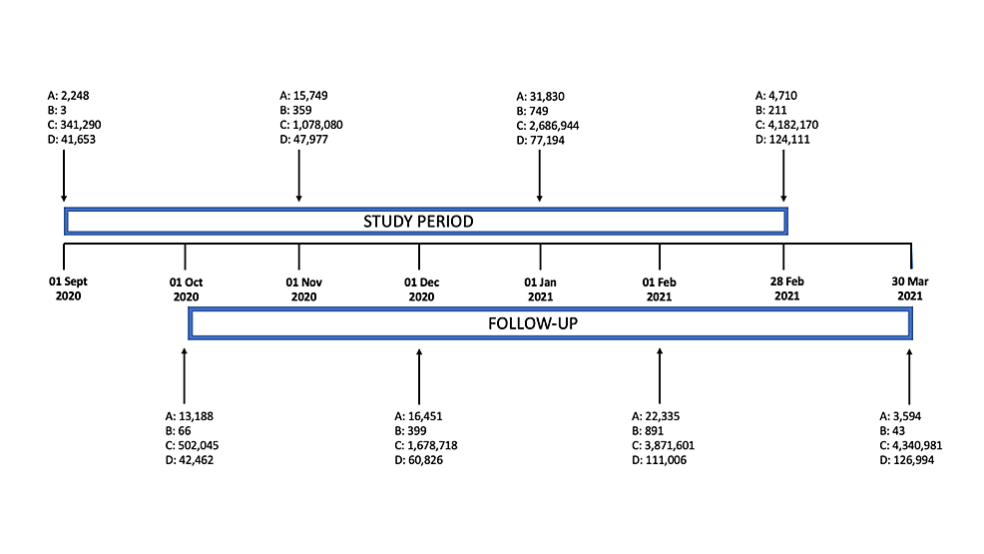
Timeline of the study period and follow-up with UK COVID-19 cases and deaths (A) Total number of daily cases, (B) total number of daily deaths, (C) total number of cases since the start of the pandemic, and (D) total number of deaths since the start of the pandemic. Source: Coronavirus UK government database.

Figure 1 demonstrates the study timeline and follow up and its association with COVID-19 cases and deaths in the UK.

Outcomes

Primary Outcome

The primary outcome was 30-day post-operative COVID-19 mortality.

Secondary Outcome

The secondary outcomes included: (i) 30-day post-operative non-COVID-19 mortality, (ii) 30-day post-operative complications, ​​​​​​​(iii) 30-day post-operative operations, ​​​​​​​(iv) 30-day post-operative readmissions, ​​​​​​​and (v) 30-day COVID-19 symptoms.

Data

Data were collected on patient demographics (age and gender) and co-morbidities (hypertension, ischemic heart disease, diabetes, chronic kidney disease, asthma, chronic obstructive pulmonary disease, heart failure, stroke, previous history of cancer). Operative details included cancer waiting time (CWT) where appropriate and operation details. Operations were classified into minor, intermediate and major/complex as per the NICE guidelines [16]. Thirty-day post-operative follow-up data included re-admissions, complications, COVID-19 symptoms and mortality (COVID-19 related and non-COVID-19 related). Complications were classified as per the Clavien-Dindo classification [17]. All the data were formatted in a datasheet using Microsoft Excel (Microsoft® Corp., Redmond, WA). Categorical variables were presented as number and percentage. Continuous variables were presented as median and interquartile ranges.

The study was registered locally as an audit. Ethics committee approval was not required as it was a retrospective non-interventional study.

## Results

During the study period, 331 patients underwent a surgical procedure. Of these, 162 (48.94%) were males and 169 (51.05%) were females. The median age was 55 years (interquartile range, IQR 41-67 years). Most of the patients were American Society of Anaesthesiologists (ASA) grade 2 (n=177, 53.47%), followed by ASA 3 (n=76, 22.96%) and ASA 1 (n=73, 22.05%). The most common co-morbidities included hypertension (n=87, 26.28%) and diabetes mellitus (n=32, 9.66%). Approximately 20% had a previous history of cancer (n=66) while 14% had been shielding earlier in the year (n=47; Table 1).

**Table 1:** Patient demographics (n=331)

Variable	Number n (%)
Gender	
Male	162 (48.94)
Female	169 (51.05)
Co-morbidities	
Hypertension	87 (26.28)
Ischemic heart disease	18 (5.43)
Diabetes mellitus	32 (9.66)
Chronic kidney disease	10 (3.02)
Asthma	31 (9.36)
Chronic obstructive pulmonary disease	9 (2.71)
Heart failure	4 (1.20)
Stroke	10 (3.02)
Previous history of cancer	66 (19.93)
Shielding earlier in the year	47 (14.19)
ASA grade	
ASA 1	73 (22.05)
ASA 2	177 (53.47)
ASA 3	76 (22.96)
ASA 4	5 (1.51)

**Figure 2:**
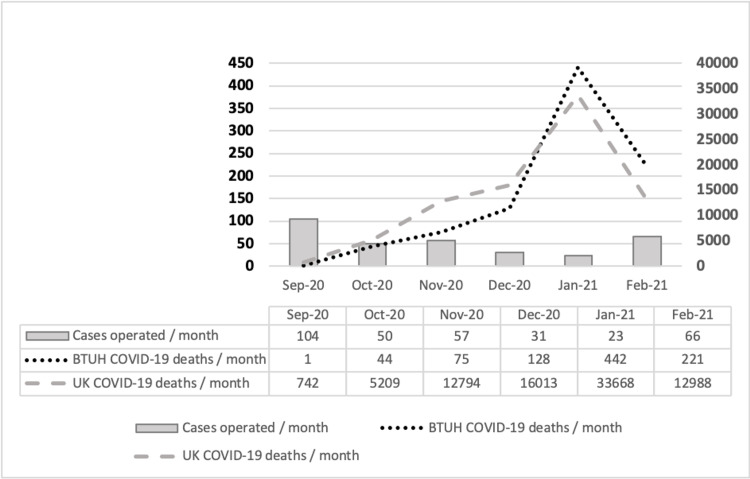
Breakdown of cases operated per month in comparison with local (BTUH) and national (UK) COVID-19 deaths BTUH: Basildon and Thurrock University Hospital

Over the six-month study period, the proportion of cases operated on was highest within the first month (September 2020, n=104) and the least number of cases were operated in the second-last month (January 2021, n=23). This coincided with the beginning and the peak of the second wave of COVID-19 in the UK. Figure 2 depicts the corelation between the number of cases operated per month during the study period with the local hospital and national deaths in the UK.

Twenty-seven percent (n=91) of patients were on the CWT list. With regards to the operative severity as per the NICE guidance, most of the operations were classed as major (51%), followed by intermediate (32%) and minor (16%; Table 2). A total of 148 patients (44.71%) underwent laparoscopic surgery while six (1.81%) had laparoscopic converted to open operation.

**Table 2:** Operation classification as per the NICE guidance NICE: National Institute for Health and Care Excellence

Variable	Number n (%)
Minor	53 (16.01)
Intermediate	107 (32.32)
Major/complex	171 (51.66)

Sixty-seven percent (n=224) of the operations were day case procedures whilst 16% (n=55) stayed for greater than three days (Table 3). There was no 30-day mortality (COVID-19 or non-COVID-19 related) in this study. The 30-day readmission and complication rate were 4% (n=14) and 6% (n=21), respectively (Table 4). Most of the complications were Clavien-Dindo grade 2 (n=10, 3.02%). There were five (1.50%) grade 3b complications requiring re-operation (Table 5).

**Table 3:** Length of stay

Variable	Number n (%)
Day case	224 (67.67)
1 day	30 (9.06)
2 days	10 (3.02)
3 days	12 (3.62)
Greater than 3 days	55 (16.61)

**Table 4:** Thirty-day follow-up data

Variable	Number n (%)
Mortality	
COVID-19 related	0 (0)
Non-COVID-19 related	0 (0)
Readmissions	14 (4.22%)
Complications	21 (6.34%)

**Table 5:** Clavien-Dindo grade and complications

Variable	Number n (%)
Grade 1	5 (1.50)
Urinary retention	1 (0.30)
Collection	1 (0.30)
Hematoma	1 (0.30)
Ileus	2 (0.60)
Grade 2	10 (3.02)
Wound infection requiring oral antibiotics	4 (1.20)
Wound infection requiring intravenous antibiotics	1 (0.30)
Intra-abdominal collection requiring intravenous antibiotics	1 (0.30)
Chest infection requiring antibiotics	2 (0.60)
Ileus requiring total parenteral nutrition	1 (0.30)
Dehydration and acute kidney injury requiring intravenous fluids	1 (0.30)
Grade 3a	1 (0.30)
Post-cholecystectomy jaundice requiring ERCP	1 (0.30)
Grade 3b	5 (1.50)
Parastomal hernia following anterior resection	1 (0.30)
Urinary bladder injury following inguinal hernia repair	1 (0.30)
Pelvic hematoma following anterior resection	1 (0.30)
Intra-abdominal collection following laparoscopic cholecystectomy	1 (0.30)
Wound infection following inguinal hernia repair	1 (0.30)
Grade 4	0 (0)
Grade 5	0 (0)

At the 30-day telephonic follow-up, 24 patients (7.25%) were non-contactable despite two attempts over two days. Of the remaining 307 patients (92.74%) who responded, 18 patients (5.86%) developed COVID-19-related symptoms. The most common symptoms were fatigue (n=7, 2.28%) and body aches (n=5, 1.62%). The median number of days after surgery for the development of COVID-19-related symptoms was 14 days (IQR 9 -19 days; Table 6). All of these 18 patients underwent a COVID PCR swab test. Only one patient tested positive, 26 days after a day case procedure.

**Table 6:** Thirty-day follow-up COVID-19 symptoms (n=307)

Variable	Number n (%)
COVID-19 symptoms	18 (5.86)
Fever	4 (1.30)
Cough	4 (1.30)
Shortness of breath	3 (0.97)
Rigors	3 (0.97)
Fatigue	7 (2.28)
Body ache	5 (1.62)
Loss of smell	1 (0.32)
Loss of taste	3 (0.97)
Number of days after surgery for the onset of symptoms (median)	14 (IQR 9-19)

## Discussion

This study found that a COVID-19-free surgical pathway, namely ‘Green Zone’ elective operating, resulted in no mortality or post-operative respiratory complication. The study period coincides with the second peak of the pandemic in the UK. We demonstrated that planned elective surgery for clinically urgent and cancer cases could be continued provided patients were appropriately counselled and adequate systems of safe operating were available. The use of a dedicated GZ in an acute trust relies on robust pathways in order to perform elective surgery safely, whilst continuing the acute activity across the trust with non-elective non-isolated patients.

In our study, 5.5% (n=18) of patients underwent COVID PCR swab testing within the 30-day post-operative period with only one positive case, 26 days after the index operation. It is quite possible that some patients may have had asymptomatic COVID infection, so the actual rate may be greater than what we have reported. However, the key thing was that all patients who had COVID-related symptoms were swabbed in the post-operative period. In order to reduce the bias regarding follow-up with patients presenting to other hospitals post-discharge, 30-day telephone consultation helped to mitigate this.

The current study is clinically more relevant to the current practice as it was conducted during the second wave of COVID-19 in the UK. Most or all elective activity came to a halt in the first wave. However, this was different in the second wave. Some hospitals relied on access to the independent sector to establish safe pathways, whilst others like us developed similar but robust pathways within the acute hospitals. It was a constantly evolving situation with new evidence and guidance becoming available every day. This required a dynamic approach whilst maintaining adequate patient safety. Use of PPE, pre-operative testing, social distancing, and self-isolation in the pre-operative period were constantly reviewed. In the initial part of the study, as per the national guidelines, patients were advised to isolate for 14 days which later changed to seven and is currently three days. A recently published study concluded that longer periods of isolation showed no reduction in the risk of post-operative pulmonary complications [18].

The overall volume of surgical activity in the UK was 33.6% lower than expected within a typical year [19]. This mainly represents the cancellation of the elective activity due to the pandemic. Approximately 2.4 million surgical procedures will be outstanding by the end of 2021, representing more than six months of normal surgical activity [19]. Dealing with the backlog will be a major challenge globally [20]. This will require total re-organisation of the services. GZ operating will become the norm. However, whilst increasing such efforts are required to clear the backlog, physical and mental burnout of the staff should be considered as well.

Limitations of the current study include being a single centre and a limited sample size. However, even with these limitations, it will be a helpful guide in planning and drafting safe elective surgery pathways in the future.

## Conclusions

This study highlights the usefulness and clinical effectiveness of the COVID-free surgical pathway (GZ) in the UK. In order to continue elective surgery during the pandemic, careful consideration should be given to appropriate patient selection, pre-operative isolation, pre-operative COVID PCR swab testing, adequate PPE, a safe theatre environment, and post-operative management. The results of the study demonstrate that elective surgery can be safely performed during the COVID-19 pandemic provided robust COVID-free surgical pathways are in place. Adherence to these pathways is the key to continue elective surgery and clear the massive backlog created by the COVID-19 pandemic.

## Figures and Tables

**Figure 3:**
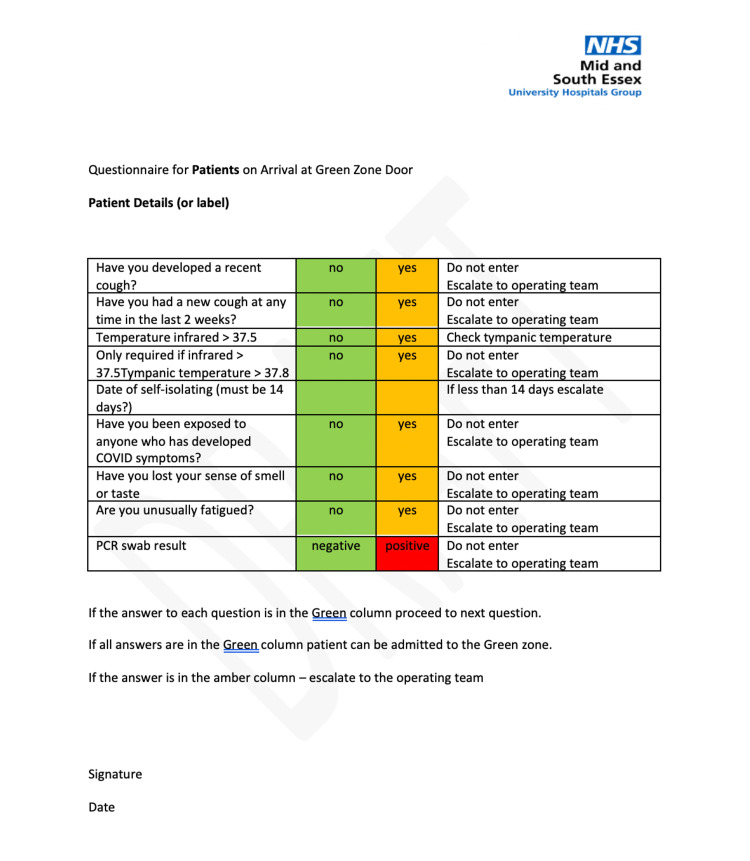
Questionnaire for patients

**Figure 4:**
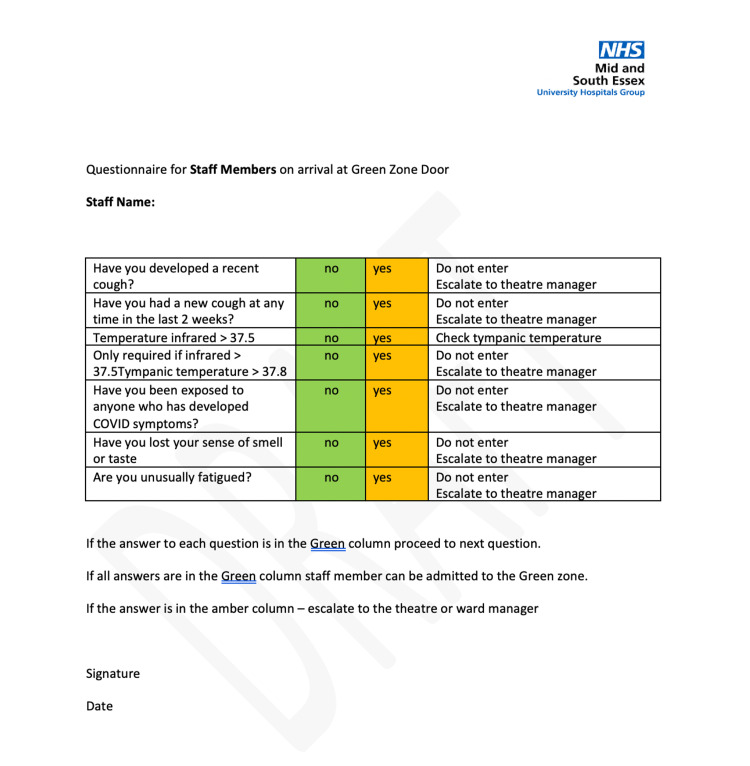
Questionnaire for staff
